# Antiviral Stewardship in Transplantation

**DOI:** 10.3390/v16121884

**Published:** 2024-12-05

**Authors:** Sruthi Bonda, Sonya Trinh, Jonathan Hand

**Affiliations:** 1 Department of Infectious Diseases, Ochsner Medical Center, New Orleans, LA 70115, USA; 2 Ochsner Clinical School, University of Queensland School of Medicine, New Orleans, LA 70115, USA

**Keywords:** antimicrobial stewardship, antiviral stewardship, cytomegalovirus, respiratory syncytial virus

## Abstract

Though antimicrobial stewardship programs (ASPs) are required for hospitals, the involvement of transplant recipients in programmatic interventions, protocols, and metrics has historically been limited. Though there is a growing interest in studying stewardship practices in transplant patients, optimal practices have not been clearly established. A component of ASPs, antiviral stewardship (AVS), specifically targeting cytomegalovirus (CMV), has been more recently described. Understanding AVS opportunities and interventions is particularly important for transplant recipients, given the morbidity and mortality associated with viral infections, challenging clinical syndromes, ultrasensitive molecular diagnostic assays, antiviral resistance, and costs of viral disease and medications, as well as antiviral drug toxicities. This review highlights opportunities for AVS for CMV, EBV, HSV, VZV, SARS-CoV-2, respiratory syncytial virus, and BK polyomavirus in transplant patients.

## 1. Introduction

Antimicrobial stewardship programs (ASPs) promote the appropriate use of antimicrobials with the goal of improving patient outcomes, decreasing unintended adverse effects, and reducing the development of multidrug-resistant organisms (MDROs). Among solid organ transplant (SOT) and hematopoietic-cell transplant (HCT) recipients, antimicrobial stewardship is critical to preserve the efficacy of antimicrobials given the increased risk of both severe and recurrent infections related to underlying immunosuppression. Furthermore, the appropriate use of antimicrobials is essential to avoid drug interactions with immunosuppressive agents and prevent adverse effects on bone marrow, organ, and graft function [1,2].

Historically, ASPs have focused on inpatient settings to provide general guidance on targeted antimicrobial therapy for infections by organ system. This generalized approach is a challenge to apply to transplant recipients as each patient has a unique set of risk factors to consider, including history of colonization with MDROs, viral serostatus, timing since transplant, type of graft, and intensity and duration of immunosuppression. Transplant ASPs build on the goal of optimizing antimicrobial use in both the outpatient and inpatient settings to improve clinical outcomes and decrease unintended adverse effects while considering each patient’s unique set of infectious risk factors [1].

While ASPs have primarily addressed the appropriate use of antibacterial and antifungal agents, antiviral stewardship (AVS) in transplant recipients is a growing area of research [3,4]. Though less frequently problematic in immunocompetent patients, common viruses encountered after transplant include cytomegalovirus (CMV), Epstein–Barr virus (EBV), herpes simplex virus (HSV), varicella-zoster virus (VZV), human-herpes virus 6, 7, 8 (HHV-6, HHV-7, HHV-8), and BK polyomavirus (BKPyV). Additionally, widespread community-acquired respiratory viruses such as influenza, respiratory syncytial virus (RSV), adenovirus (AdV), and SARS-CoV-2 may lead to significant morbidity and mortality in immunocompromised patients.

Unfortunately, data evaluating efficacy of antiviral treatment for many of the above viruses that impact transplant recipients are limited to underpowered, uncontrolled studies with heterogenous patient populations, and treatment recommendations are based on expert opinion [5,6,7,8,9]. Additionally, low-level viral replication and detection may not be associated with clinical disease or complications in some populations of transplant recipients (e.g., CMV, HHV-6, BKPyV). In many instances of low-level viral replication, these viruses do not need to be treated. However, the lack of validated viral detection thresholds and heterogeneity of net-immunosuppression across transplant populations (SOT/HCT) further increases the variability of practice.

Many available antivirals commonly used for these viruses may cause significant patient toxicity (ganciclovir, foscarnet, ribavirin, cidofovir). Moreover, prolonged use of valganciclovir/ganciclovir (vGCV/GCV) is associated with (val)ganciclovir-resistant CMV, and emergence of resistance to newer agents like maribavir and letermovir has been described [10,11,12,13]. Furthermore, the overuse of antivirals may limit the development of viral-specific cell-mediated immunity leading to late-stage reactivation of viral infections in the setting of increased immunosuppression [14].

Though lack of evidence and limited consensus guidance may lead to management challenges, antiviral drug toxicity, resistance concerns, cost, and the arrival new antivirals on the market make these agents and clinical syndromes prime targets for AVS programmatic interventions. This article will review opportunities to optimize, personalize, or avoid unnecessary antiviral therapy in SOT and HCT recipients focusing on CMV, EBV, HSV/VZV, SARS-CoV-2, RSV, and BKPyV. Though antivirals are recommended for influenza, opportunities for AVS are limited and will not be discussed.

## 2. Antiviral Stewardship for Cytomegalovirus After Solid Organ Transplantation

Cytomegalovirus remains one the most common infections after SOT and HCT, with direct and indirect effects increasing the risk for other types of infections, impacting graft function, and is associated with significant morbidity and even mortality [15]. Further, CMV continues to challenge patients and clinicians in the current era of prophylaxis, rapid molecular tests, and CMV-specific cell-mediated immunity (CMI) assays.

The most comprehensive CMV-specific stewardship program recently described by Jorgensen et al. was initiated amidst institutional concerns regarding CMV resistance, related hospital admissions, prolonged admissions, associated costs, graft outcomes, and antiviral toxicities [3]. This program was modeled on traditional ASPs and was supported by a multidisciplinary group of clinicians from pharmacy, transplant ID, and abdominal transplant physicians and surgeons, and relied heavily on prospective audit and feedback [4]. Consensus guidelines were created for prophylaxis and treatment agents, dosing and duration, and screening and diagnosis incorporating CMV cell-mediated immunity (CMI) testing to dictate the need for further surveillance. This CMV stewardship bundled intervention significantly decreased the valganciclovir days of therapy (DOT) as well as the rate of ganciclovir-resistant CMV over time [3].

### 2.1. CMV Prevention

Prophylaxis and pre-emptive therapy (PET) are acceptable CMV prevention strategies, with PET mostly utilized after HCT [15]. However, centers may consider PET as a stewardship-concordant strategy in SOT, with recent data in high-risk liver transplant recipients supporting better CMV-specific outcomes, including CMV immunologic endpoints and 5-year survival [16,17,18].

Though historically vGCV has been the most widely used antiviral for primary CMV prophylaxis after SOT, a recent randomized, multicenter phase 3 trial evaluated kidney transplant recipients with high-risk CMV serostatus (D+/R−), comparing primary prophylaxis with letermovir (with acyclovir) to vGCV, and found that letermovir was noninferior to vGCV, with lower rates of leukopenia and neutropenia [19]. Real-world experience with letermovir primary prophylaxis in other SOT populations (e.g., heart, lung transplant) has been described in small retrospective studies and may be helpful in patients unable to obtain or tolerate vGCV [20,21]. The benefits of letermovir prophylaxis need to be weighed against other factors including cost, access, adverse effects, and drug interactions [22,23,24]. Reported adverse effects related to letermovir include nausea, vomiting, diarrhea, edema, and atrial fibrillation or flutter [22,25]. There are multiple drug interactions to consider when using letermovir as it decreases voriconazole and increases tacrolimus, sirolimus, and cyclosporine levels [23,24]. Larger studies, including cost-effectiveness analyses, are needed to understand and refine ideal letermovir prophylactic strategies in SOT. Until more is understood, targeting inappropriate letermovir use may be an AVS opportunity for many transplant centers. Importantly, letermovir does not have activity against HSV or VZV, and prophylactic agents active against these viruses, e.g., (v)ACV, should be added if indicated.

Preventing CMV recurrence after therapy may be challenging in SOT recipients, and some centers routinely give secondary antiviral prophylaxis to all transplant recipients. Though no prospective, randomized trials have been carried out to evaluate the role of secondary prophylaxis to prevent CMV recurrence, multiple retrospective studies have demonstrated no benefit [26,27,28]. Further, national and international consensus guidelines recommend against the routine use of secondary prophylaxis for all patients [15,27]. These recommendations, combined with the potential antiviral toxicity, resistance, and cost, make secondary prophylaxis an ideal AVS target to address.

### 2.2. CMV Treatment

The currently available antiviral drugs for the treatment of CMV infection and disease are limited by numerous adverse effects. First-line therapy, vGCV, and GCV have unintended myelosuppressive effects, which is associated with poor outcomes in SOT [29]. Furthermore, as their administration requires dose adjustments based on renal function, exposure to subtherapeutic levels leads to the development of drug-resistant CMV [15]. Foscarnet is a recommended second-line agent for CMV treatment if patients are intolerant to vGCV/GCV, or it may be used for ganciclovir-resistant viruses. Lastly, cidofovir is associated with nephrotoxicity, electrolyte wasting, and neurotoxicity and should be avoided after SOT if possible [30].

Maribavir, a UL97 protein kinase inhibitor with a good safety profile, is a newer option for CMV in SOT. Maribavir is approved for the treatment of refractory/resistant (R/R) CMV infection or disease in SOT and HCT recipients. In the SOLSTICE trial, a phase 3, open-label study of 352 HCT and SOT recipients with R/R CMV randomized to maribavir or investigator-assigned therapy (IAT; valganciclovir/ganciclovir, foscarnet, cidofovir), maribavir was significantly more effective at CMV viremia clearance and symptom control along with fewer treatment discontinuations due to adverse effects compared to IAT [31]. Small, single-center reports of maribavir use in SOT have been published but defining indications and understanding utilization are programmatic AVS responsibilities [32]. Importantly, maribavir does not have activity against HSV or VZV, and prophylactic agents active against these viruses, e.g., (v)ACV, should be added if indicated.

Intravenous (IV) to oral (PO) conversion of antimicrobials is a main function of ASPs to avoid complications of intravenous therapy and decrease length of hospital stay [33]. With strong evidence from the VICTOR trial establishing noninferiority of valganciclovir compared to IV ganciclovir for the treatment of CMV, IV to PO conversion of valganciclovir is an additional opportunity for AVS in SOT [34]. Though case reports of antiviral therapeutic drug monitoring (TDM) have been described in SOT recipients, the lack of test availability and clinical guidance for therapeutic targets make this strategy an exciting area for potential future investigation [35].

### 2.3. Laboratory Opportunities

Working with the local laboratory and transplant team to establish and protocolize diagnostic and screening guidance and virologic thresholds for CMV treatment may be a diagnostic stewardship opportunity for AVS programs [15]. An outpatient, pharmacy-led intervention consisting of real-time CMV PCR surveillance and result notification coupled with drug therapy optimization recommendations resulted in more patients with CMV clearance and lower peak CMV viral loads [36]. Additionally, appropriate CMV resistance testing (UL97 and UL54) should only be routinely sent if DNAemia persists or increases, or if CMV-related clinical symptoms do not resolve after at least 2 weeks of appropriately dosed (v)GCV therapy [27]. Though the routine clinical role of CMV CMI testing may not be solidly established, opportunities to assess risk and personalize treatment for primary and secondary prophylaxis show promise as a future AVS tool [37,38]. Unfortunately, widespread protocolization and recommendations for testing are further challenged by the lack of commercial availability of certain CMV CMI assays (i.e., QuantiFERON-CMV) in the US. Antiviral stewardship opportunities and challenges in CMV in SOT are summarized in Table 1. 

## 3. Antiviral Stewardship for Cytomegalovirus After Hematopoietic Stem Cell Transplant

### 3.1. CMV Prevention

Though CMV is a frequently encountered viral infection after allo-HCT associated with significant morbidity and mortality, to our knowledge there has been no comprehensively described AVS or CMV stewardship program described in the HCT population [39]. The American Society for Transplantation and Cellular Therapy (ASTCT) guidelines recommend two complementary strategies for CMV prevention in CMV seropositive patients after allo-HCT in the early post-engraftment period: CMV monitoring for pre-emptive therapy (PET) and chemoprophylaxis with letermovir [40]. In a landmark phase 3, double-blinded, randomized control trial of 495 CMV seropositive allo-HCT recipients by Marty et al., patients on letermovir prophylaxis through to week 14 had a significantly lower risk of clinically significant CMV infection compared to the placebo group at the week-24 follow-up [22]. The impact of letermovir on the prevention of CMV infection and mortality was even more pronounced among patients at high risk for CMV reactivation [22].

An additional factor to consider with the use of letermovir is the potential delay in the development of CMV-specific T-cell-mediated immunity (CMV-CMI) leading to late-stage CMV reactivation after discontinuation of prophylaxis. In the landmark trial by Marty et al., clinically significant CMV infection was reported in 10% of all patients and 20% of patients at high risk for CMV infection at the 24-week follow-up, 10 weeks after letermovir prophylaxis was discontinued [22]. Similar findings were demonstrated in a phase 3, multicenter, double-blinded, randomized control trial of extended-duration letermovir prophylaxis in 220 CMV seropositive allo-HCT recipients at high risk of late CMV infection by Russo et al. [25]. Extending letermovir prophylaxis from day 100 to day 200 was safe and effective in reducing the risk of clinically significant CMV infection; however, there was no significant difference in the incidence of clinically significant CMV reactivation by the 48-week follow-up, 20 weeks after letermovir prophylaxis was discontinued [25]. The late-stage CMV reactivation in both trials was likely related to delays in the development of CMV-CMI. Zamora et al. demonstrated that patients on letermovir prophylaxis had decreased CMV-specific T-cell responses to multiple CMV antigens (immediate early-1 and phosphoprotein 65) compared to the PET group, possibly related to the suppression of CMV replication and decreased exposure to CMV antigens [14].

A stewardship initiative to optimize letermovir use after allo-HCT involves targeting chemoprophylaxis for patients at high risk for CMV infection. High-risk patients are defined as CMV seropositive allo-HCT recipients having one or more of the following criteria: mismatched donor, haploidentical donor, use of umbilical cord blood as stem-cell source, and the use of ex vivo T-cell depleted grafts [22,25,41]. A recent cost analysis comparing letermovir prophylaxis and PET through day +180 after allo-HCT found that letermovir was associated with significant reductions in CMV-related admissions and decreased the median total cost of care in high-risk CMV patients [42]. Among patients at low risk for CMV reactivation not meeting any of the above criteria, CMV monitoring with PET may be a more beneficial preventative strategy to allow exposure to CMV antigens for the development of CMV-CMI.

Building on AVS strategies for CMV prevention in SOT recipients by Jorgenson et al., a proposed clinical algorithm involves routine monitoring of CMV-CMI in the early post-engraftment period to distinguish between allo-HCT recipients with ongoing risk of developing CMV infection and those who are protected [3]. The REACT study demonstrated that allo-HCT patients with high CMV-CMI, as determined by a peptide-based enzyme linked immunospot (ELISPOT) CMV assay, were less likely to develop clinically significant CMV infection [43]. Once CMV-CMI develops during routine monitoring after allo-HCT, CMV prevention strategies, including letermovir prophylaxis and CMV monitoring with PET, can likely be discontinued.

### 3.2. CMV Treatment

Similarly to SOT, the available therapies for CMV infection and disease in HCT are also limited by numerous adverse effects. The myelosuppressive effects of vGCV/GCV may require support with granulocyte colony-stimulating factor and are generally avoided during the pre-engraftment stage, and underdosing in HCT is a risk for development of drug-resistant CMV [44]. Foscarnet is recommended for CMV treatment during the pre-engraftment stage, but toxicities are also seen in HCT patients [45]. Cidofovir, reserved for third-line therapy, should be avoided when possible [30].

Extrapolating from the results of the VICTOR trial, many subsequent studies have demonstrated that valganciclovir has equivalent clinical efficacy to ganciclovir for CMV pre-emptive therapy in HCT recipients [34,44,46]. Even in the setting of mild intestinal graft-versus-host-disease (GVHD), valganciclovir achieves equivalent serum levels to ganciclovir [47]. Therefore, intravenous GCV to oral vGCV conversion may also be a target for AVS activities in HCT recipients.

Based on the results of the SOLSTICE trial, maribavir is a promising agent for CMV PET after allo-HCT [31]. Unfortunately, maribavir may not be as effective as vGCV for the treatment of first asymptomatic CMV infection after allo-SCT. In the AURORA trial, a multicenter, double-blind, phase 3 study of 547 HCT recipients with first asymptomatic CMV infection randomized to maribavir or valganciclovir, maribavir did not meet the primary endpoint of noninferiority for CMV viremia clearance (maribavir 69.9%, valganciclovir 77.4%) at week 8. However, patients treated with maribavir maintained CMV clearance without tissue-invasive disease at week 16 post-treatment follow-up. The maribavir group was less likely to develop neutropenia and require treatment discontinuation from adverse effects [48]. Using hospital LOS data from the SOLSTICE trial and modeling CMV-related costs from the published literature, a recent US-based analysis led by maribavir’s sponsor estimated that maribavir was likely associated with cost-savings when compared to IAT for R/R CMV [49]. Additional studies are needed to determine the role of maribavir in the management of CMV infection in allo-HCT recipients.

CMV adoptive T-cell therapy is another promising treatment for the management of CMV infections, particularly in the setting of refractory and resistant CMV disease after HCT. Several nonrandomized clinical studies have demonstrated the safety and efficacy of donor-derived or third-party CMV-specific T-cells (CTLs) [50]. Unfortunately, CTLs require a lengthy and expensive process of identifying HLA-matched CMV seropositive donors with subsequent isolation of CMV-specific T-cells for transfusion. Recent advances include the development of “off-the-shelf” third-party CTLs banks [51,52]. While CMV cellular therapies are currently limited by cost and availability, their widespread use could help avoid adverse drug effects and restore CMV immunity to prevent late-stage reactivation. However, the role of these therapies is currently uncertain, and experiences should be reported as further studies are needed. Antiviral stewardship challenges and opportunities in CMV after HCT are summarized in Table 2.

## 4. Antiviral Stewardship for Respiratory Syncytial Virus

RSV is a frequently encountered community-acquired respiratory virus in transplant recipients associated with significant morbidity and mortality in immunocompromised hosts [53,54]. Among allo-HCT and lung transplant recipients, RSV upper respiratory infections (URIs) are more likely to progress to lower respiratory tract disease (LRD) with resulting respiratory failure and death [55]. Lung transplant recipients can also suffer from chronic sequelae after severe RSV infections as immune dysregulation can lead to chronic lung allograft dysfunction [56]. Ribavirin is a nucleoside analogue with in vitro activity against RSV. As there are no large prospective randomized double-blinded clinical trials on the safety and efficacy of ribavirin, there is no consensus on the optimal treatment of RSV in HCT and SOT recipients [53,54]. Based on data from observational studies, current clinical guidelines recommend considering the use of ribavirin in severely immunocompromised patients where treatment may reduce progression to LRD and death [53,54].

An important AVS initiative is reserving the use of ribavirin for patients who would receive the most clinical benefit from treatment as the drug is associated with hematologic toxicities including anemia, hemolytic anemia, neutropenia, and lymphopenia [57]. Among allo-HCT recipients, patients who are severely immunocompromised when presenting in the earliest stages of RSV URI may benefit the most from ribavirin therapy. Shah et al. developed the Immunodeficiency Scoring Index (ISI)-RSV that stratified allo-HCT recipients into three risk groups (low, moderate, and high) based on six immunodeficiency markers immediately available on routine bloodwork: age ≥ 40 years, absolute neutrophil count (ANC) < 500/μL, absolute lymphocyte count (ALC) < 200/μL, myeloablative conditioning regimen, acute or chronic GVHD, corticosteroids, and recent or pre-engraftment allo-HCT. The study found that in a cohort of 237 allo-HCT recipients, those with high risk based on the ISI-RSV benefited the most from ribavirin when administered during the URI stage, and they had the highest risk of progression to LRD and death when ribavirin was not initiated [58].

An AVS initiative to optimize ribavirin use identifies allo-HCT recipients early in the RSV disease course and reserves ribavirin for patients determined to be at high risk for severe disease. For a timely diagnosis of RSV, all allo-HCT recipients presenting in the outpatient or inpatient setting with URI symptoms would immediately be tested using a multiple polymerase chain reaction (PCR) respiratory viral panel. The decision to treat would be based on the patient’s risk for progression to LRD using the ISI-RSV. Additional risk factors for progression from URI to LRD to consider when deciding to initiate ribavirin include history of smoking, total body irradiation > 1200 cGy, use of a mismatched or unrelated donor, multiple transplantations procedures, prior exposure to antibiotics, and serum glucose > 200 mg/dL [53]. Transplant centers can consider incorporating these risk factors into treatment algorithms to assist with clinical decision making.

Another AVS opportunity involves replacing aerosolized ribavirin with the oral formulation for the treatment of RSV in severely immunocompromised hosts. The initial studies demonstrating a potential clinical benefit of ribavirin used the aerosolized formulation for the treatment of RSV [59,60,61]. However, there are many drawbacks to aerosolized ribavirin, including the risk of bronchospasm, pulmonary edema, dyspnea, and impaired ventilation and oxygenation in the patient. An additional limitation to ribavirin includes its potential teratogenicity to healthcare providers exposed to the drug. Administration of aerosolized ribavirin requires a scavenging tent, a process that is challenging for the respiratory therapist and uncomfortable for patients who may already be experiencing respiratory distress [62]. The decision to initiate treatment with aerosolized ribavirin is challenging as clinicians must weigh the potential risks with unknown clinical benefit given the minimal evidence supporting its use.

Subsequent research has demonstrated that oral and aerosolized ribavirin formulations have similar efficacy in the treatment of RSV for both allo-HCT and lung transplant recipients [63,64]. A retrospective cohort study of 124 allo-HCT recipients with RSV infection found that the rate of progression to LRD and 30-day mortality were similar among those treated with oral and nebulized ribavirin [63]. In a retrospective study of 52 lung transplant recipients with symptomatic RSV infection, oral ribavirin was found to be an effective and well-tolerated alternative to inhaled ribavirin with associated cost savings and reduced length of hospital stay [64]. Restricting adult hospital formularies to oral ribavirin may decrease healthcare costs and prevent adverse effects in patients and healthcare workers.

The US Food and Drug Administration has approved multiple safe and effective vaccines for the prevention of RSV LRD in adults ≥ 60 years: adjuvant monovalent vaccine Arexvy, nonadjuvanted bivalent vaccine Abrysvo, and the mRNA vaccine mResvia [65]. Widespread immunization of immunocompromised patients at high risk for LRD is the most impactful stewardship intervention to decrease the incidence of symptomatic RSV, prevent hospitalizations, and obviate the need for ribavirin. As the vaccines clinical trials did not include immunocompromised hosts, there are no clinical guidelines on their use among SOT and HCT recipients [66,67,68]. The immunogenicity of RSV vaccine among immunocompromised hosts is an active area of research that will guide future recommendations on the optimal age of vaccine initiation and the frequency of boosters. Based on the available information, an AVS strategy to increase RSV vaccination rates involves the routine recommendation of RSV vaccines during pretransplant counseling visits to ensure that vaccines are administered prior to the onset of immunosuppression. For pre- and post-transplant patients ≤ 60 years, shared clinical decision making between the clinician and the patient is encouraged to discuss the potential benefits while weighing the possible out-of-pocket cost for the vaccine. Considerations for AVS for RSV after transplantation are summarized in Table 3.

### 4.1. Antiviral Stewardship for SARS-CoV-2

Immunosuppression has been associated with an increased risk of COVID-19-related hospitalizations in SOT recipients [69]. While a majority of HCT and SOT patients appear to have lower disease severity from infections with current variants, certain groups, such as lung transplant recipients, may still experience severe COVID-19 [70,71]. Unfortunately, resistance development on therapy has been described in immunocompromised patients [72].

Transplant recipients were largely excluded from early-pandemic, antiviral trials, and large, robust studies in transplant populations are lacking. There have been prospective and retrospective studies evaluating antiviral interventions (remdesivir, nirmatrelvir/ritonavir, molnupiravir) in transplant recipients with COVID-19 [73,74,75]. The Infectious Diseases Society of America (IDSA) treatment guidelines do not give specific antiviral treatment recommendations for transplant recipients, though currently available guidance from transplant experts is similar [76,77,78]. For patients with mild to moderate COVID-19, guideline-recommended options are remdesivir (3 days), nirmatrelvir–ritonavir, or molnupiravir, or neutralizing monoclonal antibodies and 5 days of remdesivir if patients have severe COVID-19 and are on supplemental oxygen. Though nirmatrelvir-ritonavir has been safely used in SOT and HCT recipients, ritonavir’s strong inhibition of cytochrome P450 3A4 leads to a substantial interaction with calcineurin inhibitors (CNIs), which may lead to severe toxicities [75].

The changing variant landscape and immunity conferred from prior infection and SARS-CoV-2 vaccination further challenges understanding of the most ideal patient, time, and setting of antiviral interventions. AVS initiatives for SARS-CoV-2 may focus on institutional protocols supporting clinicians to understand an individual SOT/HCT recipient’s risk of severe disease and subsequent need for antivirals in low-risk, vaccinated patients with mild symptoms. Additionally, ensuring appropriate durations of remdesivir based on disease severity (3 days mild–moderate; 5 days for severe) may be another opportunity for AVS monitoring. Working with transplant pharmacy teams to support management and patient education regarding nirmatrelvir–ritonavir CNI interactions may also be beneficial. To avoid unnecessary antivirals, AVS for SARS-CoV-2 should include diagnostic stewardship, avoiding screening of asymptomatic patients in most circumstances in the pre- and post-transplant setting. The use of immunomodulatory agents for patients with severe COVID-19 may also be an opportunity for AVS support, though this is beyond the scope of this review. Considerations for AVS for SARS-CoV-2 after transplantation are summarized in Table 4.

### 4.2. Antiviral Stewardship for HSV/VZV

After SOT, most recipients receive vGCV prophylaxis, which generally provides protection against HSV and VZV reactivations. In some situations, (v)ACV may be given instead of vGCV (toxicity, cost, CMV D-/R-) for a recommended duration of at least 1 month or after episodes of rejection [79]. After HCT, HSV risk is highest in the early post-transplant period. ACV prophylaxis decreases HSV and VZV reactivation [80]. The National Comprehensive Cancer Network (NCCN) recommends HSV prophylaxis for a minimum of 2 months after alemtuzumab and until CD4 ≥ 200 cells/mcL and VZV prophylaxis for at least 1 year after allogeneic HCT [81].

Optimizing institutional protocols for prophylaxis and treatment and attempting to monitor prescription indications for (v)ACV is an additional opportunity for AVS. Further, in coordination with transplant pharmacy clinicians, AVS should work to ensure that patients who are prescribed letermovir for CMV prophylaxis (after SOT or HCT) and/or maribavir for refractory/resistant CMV therapy are also on an HSV/VZV-active agent such as (v)ACV if they meet the criteria to receive HSV/VZV prophylaxis.

Empiric ACV for patients with presumed HSV or VZV encephalitis is commonly encountered in clinical practice. Studies including transplant and immunocompromised patients have reported decreases in ACV utilization after implementation of meningitis encephalitis panels, which may support AVS [82,83]. However, it is important to keep in mind that false-negative HSV PCR results have been reported from meningitis encephalitis panels, and HSV 1/2-specific PCR testing should be considered if concern for infection remains [84].

### 4.3. Antiviral Stewardship for EBV

In patients who have undergone SOT or HCT, EBV reactivation is common and can be associated with a variety of clinical syndromes. However, EBV-associated post-transplant lymphoproliferative disorder is the most concerning and challenging. Though monitoring EBV DNAemia is common after SOT and HCT, relevant thresholds dictating interventions and predicting PTLD across different organs transplanted, as well as variability across labs, further challenges EBV management in transplantation. EBV DNAemia monitoring for allo-HCT recipients is recommended to begin within the first month post-transplant and continue weekly for at least 4 months and longer if patients are suspected to have poor T-cell reconstitution [85]. In EBV-negative SOT recipients of EBV seropositive donors, EBV DNA monitoring is recommended weekly to biweekly in the first year after transplant [86]. In patients who develop EBV DNAemia (center-defined threshold), weekly rituximab is the first-line agent for preemptive therapy and is given weekly for 1–4 doses or until EBV DNAemia negativity and should be combined with immunosuppression reduction when possible [85]. In SOT recipients with EBV DNAemia, a reduction in immunosuppression is the first-line recommended management strategy, as data using rituximab therapy are limited [86]. EBV DNA monitoring after HCT or mismatched SOT may be an opportunity for diagnostic stewardship. Optimization of rituximab prescription may also be supported by AVS programs targeting center-specific guideline adherence in order to decrease practice variability.

While ACV and GCV have in vitro activity against lytic forms of EBV, and the lytic cycle is associated with the progression of EBV tumors, latent EBV-driven lymphoproliferation predominates in PTLD lesions [87]. However, ACV and GCV have no impact on latent EBV infection. Though some single- and multicenter retrospective studies suggest that antiviral prophylaxis reduces PTLD incidence, larger, more methodologically robust studies demonstrate otherwise [86]. Therefore, the use of antivirals, (v)ACV and (v)GCV, for prophylaxis or preemptive therapy to prevent EBV-positive PTLD is not recommended after SOT or HCT [85,86]. Similarly, there is insufficient evidence to support the routine use of antiviral therapy for the treatment of EBV-positive PTLD after SOT or HCT [85,86]. Considering that the totality of data does not support antivirals for EBV, and national and international guidelines recommend that antivirals should not be used, antiviral utilization for the prevention or treatment of EBV may be an opportunity for AVS through the creation of institutional guidance and monitoring drug utilization for this indication. If available, cellular therapy, such as EBV-specific CTLs, is recommended to prevent and treat PTLD after HCT but not after SOT, given limited data [85,86]. The role of CTLs in AVS has not yet been determined and needs further study. Emerging diagnostics, such as those testing for the Z EBV replication activator (ZEBRA) protein, identifying lytic virus, have been studied in transplant recipients, and newer strategies to inhibit EBV lytic replication are being explored [88,89]. The future role for these diagnostic and therapeutic interventions within AVS programs remains to be determined. Considerations for AVS for EBV after transplantation are summarized in Table 5.

### 4.4. Antiviral Stewardship for BK Polyomavirus in Transplantation

Lowering immunosuppression is the primary recommended management strategy for BKPyV DNAemia or related disease. Though cidofovir has in vitro activity against BKPyV, there are no prospective, well-controlled trials demonstrating clinical efficacy, and, ideally, it should be avoided in kidney transplant recipients where BKpyV is most commonly managed [90]. Recently updated international guidelines recommend against the use of cidofovir for BKPyV DNAemia or nephropathy in kidney transplant recipients [91]. Additionally, though past clinical reports of fluoroquinolones’ impacts on BK-related disease have been mixed, a more recent systematic review and two prospective RCTs found that quinolones did not prevent BKPyV DNAemia or disease and, in one study, were associated with increased rates of fluroquinolone-resistant infections [92,93,94]. Consequently, recent guidelines recommend against the use of fluroquinolones to prevent or treat BKPyV DNAemia or nephropathy after kidney transplantation [91]. Similarly, systemic cidofovir has been evaluated for the treatment of BKPyV-associated hemorrhagic cystitis in patients after HCT in small, observational studies with mixed results and should not be routinely used [5,95].

## 5. Potential Antiviral Stewardship Program Metrics

Evaluating and reporting metrics are recommended for ASPs in the general population, though transplant-specific metrics, particularly ideal AVS measures, are not as well understood [33]. Results from a 2015 survey by Seo et al. found that 23% of transplant programs that included SOT/HCT in ASPs did not track outcome metrics in these patient groups [96]. Metrics used in the comprehensive CMV stewardship program described by Jorgenson et al. included antiviral utilization, rates of GCV-resistant CMV, and hospital admission and duration for IV foscarnet [3]. AVS process metrics may focus on institutional guideline adherence, diagnostic optimization, and appropriateness of antivirals (e.g., inhaled or PO ribavirin, letermovir, and maribavir). Outcome and balancing metrics such as CMV DNAemia and disease recurrence, related hospitalizations, adverse drug events, and resistance are more resource-intensive and may be more challenging to monitor. Examples of antiviral stewardship program metrics are summarized in Table 6.

## 6. Conclusions

There are significant opportunities for AVS in transplant recipients to optimize patient outcomes. Though AVS can be easily integrated with institutional ASPs, further research on program structure, optimal interventions, and valuable metrics is needed.

## Figures and Tables

**Table 1:** Antiviral stewardship challenges and opportunities in cytomegalovirus in solid organ transplantation.

Focus Area	Challenges	Opportunities
Pre-emptive therapy (PET)	vGCV prophylaxis most common strategy. PET is resource-intensive.	PET personalized therapy, improved CMV, immunologic, mortality endpoints vs. prophylaxis in high-risk liver transplant.
Letermovir prophylaxis	Limited evidence outside of kidney transplant. Cost, availability, drug interactions.	Prophylaxis targeted to groups intolerant of vGCV, cytopenias.
Secondary prophylaxis	Recurrent DNAemia/disease may occur after therapy cessation.	Secondary prophylaxis should be avoided generally. Consider for high-risk patients with severe disease. CMI monitoring may be helpful.
Drug administration	Moderate–severe disease may require hospitalization.	IV to oral transition should be performed in most scenarios.
Therapy	vGCV/GCV with myelosuppressive effects, renal dose adjustment. Foscarnet with nephrotoxicity, electrolyte wasting.	Maribavir less myelosuppressive and nephrotoxic. Consider for R/R disease, GCV intolerance, and cytopenias.

PET: pre-emptive therapy; vGCV: valganciclovir; GCV: ganciclovir; CMI: cell-mediated immunity; R/R: refractory/resistant.

**Table 2:** Antiviral stewardship challenges and opportunities in cytomegalovirus in allogeneic hematopoietic cell transplantation.

Focus Area	Challenges	Opportunities
Letermovir prophylaxis	Delays CMV-CMI with late-stage reactivation. Cost, availability, drug interactions.	Prophylaxis targeted to groups with high risk of CMV infection. Duration of prophylaxis guided by CMV-CMI.
Drug administration	Oral vGCV absorption in setting of gastrointestinal disease and GVHD.	Transition from intravenous to oral therapy with GVHD stage ≤2.
Therapy	vGCV and GCV with myelosuppressive effects, requires renal dose adjustment. Foscarnet with nephrotoxicity, electrolyte wasting.	Maribavir less myelosuppressive and nephrotoxic. CTLs may restore CMV-CMI and prevent late-stage reactivation; role in AVS needs further definition.

CMV-CMI: cytomegalovirus T-cell-mediated immunity; GVHD: graft-versus-host disease; CTL: CMV-specific T-cells.

**Table 3:** Antiviral stewardship challenges and opportunities in respiratory syncytial virus.

Target Area	Challenges	Opportunities
Rapid diagnostics	Ribavirin therapy less effective at LRD stage.	Clinical treatment guideline for alloHCT/lung transplant presenting with URI symptoms with prompt testing using multiplex PCR respiratory viral panel.
Targeted treatment	Minimal benefit of ribavirin in low- and moderate-risk ISI-RSV patients.	Treatment algorithm reserving ribavirin treatment for alloHCT/lung transplant with high-risk patients ISI-RSV.
Drug administration	Toxicity of nebulized ribavirin to patients and healthcare providers.	Replace aerosolized with oral ribavirin in adult hospital formularies.
Vaccine	Lack of efficacy data on immunocompromised hosts.	Shared clinical decision making during pre-SOT and HCT evaluations.

LRD: lower respiratory tract disease; URI: upper respiratory tract; PCR: polymerase chain reaction; ISI: Immunodeficiency Scoring Index; SOT: solid organ transplant; HCT: hematopoietic cell transplant.

**Table 4:** Antiviral stewardship challenges and opportunities in SARS-CoV-2.

Target Area	Challenges	Opportunities
Rapid diagnostics	SARS-CoV-2 NP swab screening of asymptomatic patients may lead to over treatment.	Diagnostic stewardship limiting SARS-CoV-2 screening in asymptomatic patients in the pre- and post-transplant setting.
Targeted treatment	Unclear benefit of therapy in asymptomatic or mildly symptomatic patients.	Treatment algorithm reserving treatment for patients with significant symptoms who are high risk.
Drug administration	Remdesivir must be given intravenously.	Appropriate durations of remdesivir based on disease severity (3 days mild–moderate; 5 days severe).
Drug–drug interaction	Nirmatrelvir–ritonavir strong inhibitor of cytochrome P450 3A4, causing CNI interaction.	Educate patients and providers to avoid CNIs in SOT recipients unless close monitoring available.

NP: nasopharyngeal; SOT: solid organ transplant; CNI: calcineurin inhibitor.

**Table 5:** Antiviral stewardship challenges and opportunities in EBV.

Target Area	Challenges	Opportunities
Diagnostics	EBV DNAemia recommended in high-risk patients, no defined threshold for preemptive interventions.	Institutional protocol for EBV DNA monitoring and threshold validation.
Management	For EBV DNAemia: RIS primary strategy in SOT; rituximab primary in HCT.	Monitor appropriateness of rituximab therapy in HCT.
Antivirals	Antivirals (ACV, GCV) with in vitro activity; limited evidence for prevention or treatment of EBV/PTLD.	Monitor antivirals used to treat EBV/PTLD.
Cellular therapy	EBV CTLs recommended in HCT, not SOT.	If available, institutional protocol for CTLs; role in AVS needs further definition.

EBV: Epstein–Barr virus; RIS: reduction in immunosuppression; HCT: hematopoietic cell transplant; SOT: Solid organ transplantation; ACV: acyclovir; GCV: ganciclovir; CTLs: EBV-specific T-cells; AVS: antiviral stewardship.

**Table 6:** Examples of potential antiviral stewardship program metrics.

Process	Outcome	Balancing
Usage (DOT, LOT) Guideline adherence Intervention acceptance rate Diagnostic appropriateness (CMV PCR, CMI testing)	Infection rate: CMV DNAemia and disease. Virus-related hospitalization. Adverse drug event. Resistance rate. Time to CMV eradication.	Infection rate. Virus-related readmission. Readmission. Recurrent DNAemia/disease after treatment. Infectious mortality (CMV/RSV).

DOT: days of therapy; LOT: length of therapy; CMI: cell-mediated immunity.
